# Dana on Martin’s Bandage

**Published:** 1879-10

**Authors:** C. L. Dana

**Affiliations:** 62 W. 46th st.; New York City


### ARTICLE XIII.

NEW YORK CITY.

*Editors* CHICAGO MEDICAL JOURNAL AND EXAMINER:

In my letter from this city, published in your May number, I quoted an unfavorable opinion from the surgical staff of Roosevelt Hospital, in regard to the use of rubber bandages for ulcers of the leg. The communication seems to have found Dr. Martin in a very hyperæsthetic condition ; from which, however, I trust he was relieved by his very valuable letter in your issue for August.

Permit me to say a word in answer to it, and in defense of the Roosevelt surgeons, upon whom, I find, I have called a great deal of labored and unnecessary sarcasm.

It was not pretended in the least, that the method of treating ulcers described by me, as practiced at Roosevelt, was that advocated by Dr. Martin, nor was it assumed to be an "improvement" upon that method. It was, as I understood it, simply an experimental modification, by which it was hoped good results might be reached. The method was fully described, and no person, who had the patience to reflect, or the ability to comprehend, would infer from the statement of its poor results, that Martin's pure gum method was at all involved in the failure. The experiment was not entirely a failure either. The same dressing applied to suppurating buboes, has had remarkably good results in spite of the very shocking proximity of carbolic and boracic acids to the wounds.

Further, the remark that other surgeons, who claimed better results, had probably mistaken granulation for cicatrization, was hardly spoken *au sérieux*, nor could any mind not entirely devoid of humor, or completely preoccupied with the immense importance of pure gum, have failed to take the statement as it was meant. Fired by its apparent absurdity, however, the doctor would have us all vibrate with a noble scorn at the idea of Mr. G. W. Callender and Mr. Hutchinson not knowing a cicatrix when they see it. We consider that the doctor has undergone a very superfluous excitation of his emotional nature. No one believes, and no one seriously implied that those eminent gentlemen were not equal to the very elementary distinction referred to.

The fact is, that there has been a good deal of experimenting with the rubber bandage in this city, and much rather large talk over the wonderful results. It was this that was had in mind when the remark quoted was made.

Perhaps all this should have been explained at first, but it did not occur to me that any one would condemn Dr. Martin's pure rubber bandages, because an elaborate and fully described modification of it failed.

There have now been so many communications, extracts and discussions in regard to the bandage and Dr. Martin's views upon it, that there is little danger of any misunderstanding, or of involving him undeservedly in any failure. We would suggest, therefore, that he now let us go on and use it in peace. The medical world will have to convince itself—it will take no single individual's statement—that a whiff of carbolic acid, or a suggestion of litharge will prevent an ulcer's healing. When convinced, however, that such is the case, we all know that Dr. Martin is making the pure gum at a great personal sacrifice, and that it can be obtained of the doctor himself or his agents in London. Very truly yours,

62 W. 46TH ST. C. L. DANA.

---

DRS. F. C. HOTZ and E. J. GARDINER, have made arrangements at the Eye and Ear Infirmary for a course of lectures upon the *Physical and Functional Examination of the Eye.* This is a good opportunity for physicians interested in the study of diseases of the eye, to become thoroughly acquainted with the various methods of examining this organ. Each lecture will close with demonstrations and practical exercise with the focal illumination and ophthalmoscope; and in the testing of the refraction, accommodation, color, perception, and movements of the eye. Physicians desirous to take this course, may apply at the infirmary between two and three o'clock.

DR. BULKLEY announces a third course of free lectures on *Diseases of the Skin*, in the pathological amphitheatre of the New York Hospital, 7 West 15th St., on Wednesday afternoons, from 2:30 to 3:30 o'clock, commencing Wednesday, Oct. 8, 1879. The lectures will be didactic and clinical in character, going over the entire subject of diseases of the skin (including syphilis), and will be freely illustrated by colored plates, photographs, life-sized models, the blackboard, and abundant clinical material. The pathology, differential diagnosis, and treatment of diseases of the skin will be especially considered.

